# Development of a multichannel current-EMG system for coherence modulation with visual biofeedback

**DOI:** 10.1371/journal.pone.0206871

**Published:** 2018-11-16

**Authors:** Daniel Comaduran Marquez, Vinzenz von Tscharner, Kartikeya Murari, Benno M. Nigg

**Affiliations:** 1 Biomedical Engineering Graduate Program, University of Calgary, Calgary AB, Canada; 2 Human Performance Laboratory, University of Calgary, Calgary AB, Canada; 3 Electrical and Computer Engineering, University of Calgary, Calgary AB, Canada; University of Illinois at Urbana-Champaign, UNITED STATES

## Abstract

By means of biofeedback, neuromotor control can be modified. Recent biofeedback experiments have used the power of the electromyogram of one muscle in different frequency bands to control a two-dimensional cursor. However, the human body usually requires coherent activation of multiple muscles to achieve daily life tasks. Additionally, electromyography (EMG) instrumentation has remained the same for decades, and might not be the most suitable to measure coherent activations from pennated muscles according to recent experiments by von Tscharner and colleagues. In this study, we propose the development of a multichannel current-based EMG amplifier to use intermuscular coherence as the control feature of a visual biofeedback system. The system was used in a leg extension protocol to voluntarily increase intermuscular coherence between the vastii muscles. Results from ten subjects show that it is possible to increase intermuscular coherence through visual biofeedback. Such a system can have applications in endurance training and rehabilitation.

## Introduction

Biofeedback can be defined as a process whereby monitoring of a normally automatic bodily function is used to train someone to improve the voluntary control of such function. It is a valuable supplementary treatment and complements rehabilitation protocols to recover healthy muscle function after trauma or musculoeskeletal disease [1]. Biofeedback is often used to train neuromotor control during rehabilitation [2–9].

Neuromotor control is not limited to switching muscles on or off, it includes fine-tuned control to select the right fiber types, and activate them with precise timing. Humans use different types of muscle fibers, and it has been shown that fast and slow conducting muscle fibers contribute to low and high frequencies of the EMG spectrum [10]. Motor units (MUs) must coordinate and synchronize in such a way that they activate the muscles at the right time, for instance, while running [11]. If a pool of motor neurons within one muscle or across two muscles receives a common input from the central nervous system, the corresponding motor unit action potentials (MUAPs) will occur almost simultaneously in different areas of the muscles [12]. Synchronization of MUs generate spectral changes by grouping, and thus clustering the MUAPs, a process that affects the low frequencies of the EMG spectrum. [13]. Muscles are also controlled by varying the contributions from the motor cortex that can be seen in the coherence between electroencephalography (EEG) and EMG signals at the frequencies of the beta (12 − 30 Hz) to gamma bands (30 − 80 Hz) [14]. Thus, if there is a common input from the motor cortex to the MUs, one expects to see coherence at the frequencies where they are contributing to the EMG spectrum, in the 30 to 50 Hz frequency band. These oscillations are known as the Piper rhythm.

Recent work has shown that humans can voluntarily control some of these physiological processes, and their contributions to the EMG in such a way that the spectral properties can be used to control a human-machine interface [15, 16]. The deeper implication of this finding, as seen from a neuro-physiological point of view, is that humans can voluntarily change the interplay between physiological processes with the help of a biofeedback system. However, these studies did not indicate which one of the physiological processes mentioned above was used to change the EMG spectrum.

Synchronization of MUs seem to be important contributors to the stability of human movements that can be altered after an injury. Injuries lower the mean frequency of the power spectra of the EMG, and may cause afferent proprioceptive information to be missing. This may alter, among others, the subsequent physiological processes that determine the spectral information of the EMG spectrum [17]. Additionally, it has been shown that there are fatigue-related changes in MU synchronization of quadriceps muscles within and across legs [18]. Following the findings of Phinyomark et al., where different bands in the EMG power spectrum of a single muscle were used as a biofeedback signal [15] the question arises, can we voluntarily control the synchronization of MUs across synergistically working muscles? If so, one could monitor and train the degree of synchronization using a biofeedback process. To assess the synchronization of the muscles, magnitude square coherence (MSC) can be used. MSC is an estimate of the degree of relationship between two time-varying signals as a function of frequency [19, 20]. MSC is dependent on auto and cross-correlation spectral densities, and thus can indicate changes in the power spectrum.

There are many conceivable applications of being able to voluntarily control the synergistic activation of MUs. In our laboratory, we study the effect of injuries on synergistic activation of MUs. Our interest is understanding the physiological processes that contribute to the stability of the knee joint, and we therefore studied the vastii muscles first and will later extend the research to include the interaction of the quadriceps muscles and the hamstrings. However, the technique is new and must first be developed. To do so, it is necessary to develop instrumentation that can measure coherence reliably, and to test whether intermuscular coherence can be voluntarily modulated.

### Methodology to measure EMG

Extraction of features requires the acquisition of a reliable signal that contains the desired information. EMG signals have been acquired for decades in either a monopolar or bipolar configuration with a high impedance differential amplifier [21]. The most popular surface EMG amplifier consists of bipolar surface electrodes with an instrumentation amplifier (IA) [21]. The method is usually described for fusiform muscles, and might not be the most appropriate to quantify coherent activation of agonist pennated muscles as the common mode rejection of an IA can attenuate synchronized signals [22]. It has also been suggested that an IA allows inter-electrode currents to develop; such currents cause the signals from neighboring electrodes to show similar signals that are attenuated by the common mode rejection of the IA [22]. Lastly, signal attenuation can occur if the electrodes are misaligned to the muscle fibers, which is almost impossible to avoid when measuring from pennated muscles or during movement.

To overcome these problems, the concept of a monopolar current EMG measurement system has been previously proposed and validated [22–25]. This new method uses a transimpedance amplifier (TIA) instead of an IA. The TIA keeps the active and reference electrodes at the same potential level, eliminating inter-electrode currents, making the TIA more suitable to quantify intermuscular coherence, especially in pennated muscles [22]. The drawback using a TIA is that it is inherently single-ended, and lacks common mode rejection, which reduces noise coupled in from the environment. The existing implementation of the current-based EMG amplifier has a sub-optimal frequency response, and suffers from cross-talk when trying to measure EMG signals from different muscles simultaneously [24]. As proposed by Nann in 2014, the cross-talk occurs because the impedance at the ground electrode is larger than the impedance at the muscle electrode. To close the loop, the current returns to the body through the muscle electrode of the second amplifier. This causes the second amplifier to measure the inverted signal of the active muscle. To avoid the cross-talk, previous experiments relied on having a separate acquisition system (i.e. amplifier, data acquisition card, and computer) for each muscle [23, 24]. This setup might not be adequate for a real-time biofeedback system. Therefore, the limitations of the EMG current-amplifier in its present form have to be overcome before it can be used to quantify intermuscular coherence as an input to a biofeedback system.

### Purpose

The purpose of this study is to test whether one is able to voluntarily increase intermuscular coherence by means of a biofeedback system. The specific objectives of this study are: 1) To develop a multichannel, current-based EMG system by improving the design proposed by von Tscharner et al. [22]. 2) To test if the intermuscular coherence between the vastii muscles can be voluntarily increased using a visual biofeedback system based on the current-based EMG system.

## Methods

### Hardware development

There is a need to technically improve the current-based EMG amplifier proposed by von Tscharner et al [22]. The original current-based EMG amplifier required capacitive coupling to avoid saturation of the TIA while recording from the vastii muscles [24], as well as an isolated recording system for each muscle [23]. We chose the following specifications for the development of the new current-based EMG amplifier. First, most of the EMG signal power is contained between 10 to 500 Hz [21]. Thus, we needed a flat frequency response between 10 and 500 Hz. Second, previous experiments on the gastrocnemius medialis muscle with the current-based amplifier used a gain of 2.2 MΩ for the TIA [22]. Our experiment differed in the task performed; our task consisted of a leg extension movement with no additional load. Due to this difference in experiments, we expected a stronger signal, and therefore needed a smaller gain. We selected three gain values for the TIA. These values were chosen to change the amplification required for each subject to use most of the dynamic range of the data acquisition card (DAQ). Finally, to avoid cross-talk without using two independent recording systems, we developed an opto-coupled isolator. The opto-coupled isolator works by allowing each amplifier to have its own power supply, thus, avoiding the cross-talk. The solution of having a separate power supply to measure from multiple muscles simultaneously was recommended in previous work with the current-based amplifier [24]. However, its previous implementation is not suited to our experimental setup. All circuits were designed using Multisim 14 (National Instruments, Austin, USA). The opamp chosen was the OPA140 (Texas Instruments, Dallas, USA). This opamp was selected due to its low noise (151.9 nV integrated noise from 10 to 500 Hz); low input bias current (10 pA) and low current consumption (1.8 mA per amplifier).

#### Amplifier design

The new amplifier consisted of three stages: a TIA, a high-pass filter, and a low-pass filter. The TIA stage was configured with a three-position switch to change the gain of the amplifier, while also adjusting the feedback capacitance keeping the bandwidth constant to provide a maximum non-flatness of 0.1 dB up to 500 Hz. The resistor and capacitor values for the low, medium and high gain settings were 100 kΩ and 500 pF, 250 kΩ and 200 pF, and 500 kΩ and 100 pF, respectively.

The second and third stages are filtering stages. Both filters were designed with a Butterworth response implemented with a Sallen Key topology. This configuration was chosen to obtain a maximally flat frequency response in the band-pass region. The first filter is a fourth order high-pass filter with a cut-off of 10 Hz and a passband gain of 10; the second stage is a second order unity gain low-pass filter with a cut-off of 1 kHz. The cut-off frequency for the high-pass filter was selected from the original amplifier design [22]. The cut-off for the low-pass filter was chosen to give a maximum non-flatness of 1 dB in the overall transfer function in the 10 − 500 Hz region.

The new amplifier was implemented on a two-layer printed circuit board (PCB) measuring 20 × 30 mm^2^. The electrode, data, and power supply connectors were soldered to the PCB using right angle headers to avoid cable strain on the connections. To minimize externally induced noise (i.e. 60 Hz line frequency), we chose shielded electrode leads (AD Instruments, Dunedin, New Zealand), as well as a shielded micro USB cable to interface with the opto-coupled isolation module.

The frequency response of the amplifier was measured with a SR 760 FFT spectrum analyzer (Stanford Research Systems, Sunnyvale, USA). All the amplifier characterization measurements were performed in a Faraday cage with the system operated with ±9 V batteries to avoid power supply noise. To test the design, a transconductance amplifier (TCA) was used to convert the voltage from a function generator into a current to input into the amplifier. The integrated circuit (IC) used for the TCA is the LM13700 from Texas Instruments. Measurements for the new amplifier were done using the highest gain setting (500 kΩ). Additionally, to measure the noise of the amplifiers, input referred noise of the new and the original amplifier was calculated for frequencies between 1 Hz to 10 kHz.

#### Isolation module design

To record from multiple muscles with a single data acquisition system, an optocoupler-based isolation module was implemented. The selected optocoupler was the HCNR 200 (Avago technologies, San Jose, USA). This optocoupler was chosen because of its low non-linearity (0.01%) achieved by using two closely matched photodiodes in conjunction with a light emitting diode (LED). Feedback from one of the photodiodes is used to linearize the output light of the LED, reducing drift and non-linearities [26].

**Fig 1:**
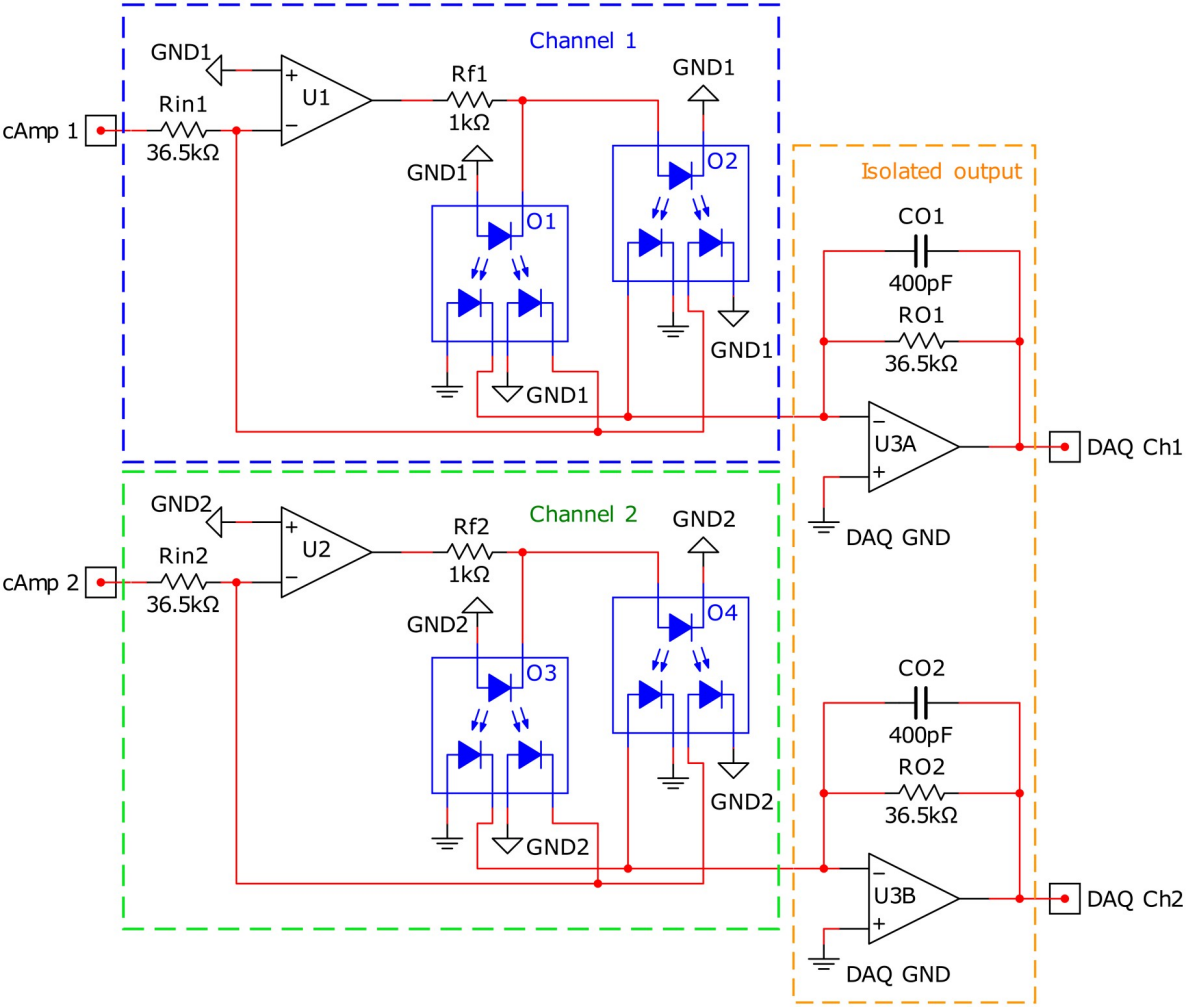
Schematic of the isolation module. The blue, green, and orange outlines mark the sections for channel 1, channel 2, and the common isolated output, respectively.

The isolation module was implemented as a bipolar input photovoltaic isolation amplifier as described in [27]. The module consisted of two channels; each channel receives the output signal from a current amplifier and shares its input-side power supply with the connected amplifier. The output of each current-amplifier (cAmp1 and cAmp2 for channel one and channel two respectively, Fig 1) is connected to a transconductance amplifier (TCA). The TCA converts the output voltage of the amplifier into a current for the optocouplers’ LEDs. Each isolation channel consists of two optocouplers, one for the positive and one for the negative side of the signal (blue and green boxes, Fig 1). When the signal is positive, current from the TCA flows through the optocoupler connected to the LED anodes (O2 and O4 for channel one and channel two respectively, Fig 1). When the signal is negative, current from the TCA flows through the optocoupler connected to the LED cathodes (O1 and O3 for channel one and channel two respectively, Fig 1). At the output of the optocouplers, there are two matched photodiodes. One of the photodiodes is fed back to the TCA to linearize the output. The other photodiode is connected to a TIA to convert the current into a voltage for digitization. The output TIA of both channels share their the power supply, and thus we can record multiple EMG signals with one data acquisition system.

The isolation module was designed with unity gain (ratio between *DAQCh*_*x*_ and *cAmp*_*x*_) and an output range of ±1 V. The resistors were calculated with equations 1 and 2 found in [27]. The values for K3 = 1 and K1 = 0.5 were taken from the HCNR200 datasheet. The feedback resistors (*Rf*_1_ and *Rf*_2_) were calculated to limit the resistor current (*IR*_*f*_) and protect the opamps *U*_1_ and *U*_2_, which have a maximum output current of 36 mA. Additionally, the capacitors (*CO*_*x*_) are used to band-limit the TIA at the outputs.

$$\begin{array}{c} \frac{DAQCh_{x}}{cAmp_{x}}=\frac{\left(K3\right)\left(RO_{x}\right)}{Rin_{x}} \end{array}$$ (1)

$$\begin{array}{c} Rf_{x}=\frac{cAmp_{x}}{\left(K1\right)\left(I_{R}f_{x}\right)} \end{array}$$ (2)

To test the isolation module, 12 EMG recordings of the left and right biceps muscle of one subject were performed in two conditions; the original amplifier without isolation, and the new amplifier with the isolation module (i.e. 6 recordings per condition). The ground electrode for each amplifier was placed on the lateral epicondyle of the radius of the corresponding arm. The subject preparation was performed following the recommendations of the Surface ElectroMyoGraphy for the Non-Invasive Assessment of Muscles (SENIAM) guidelines [28]. Each recording consisted of a single left biceps isometric contraction for a duration of 2 seconds with no added load. The intermuscular coherence (as described in the methods section) between the left and right biceps was computed as a measurement of cross-talk across the conditions.

### Experimental protocol

A biofeedback study was designed to test if the intermuscular coherence could be voluntarily increased with visual biofeedback. Ten healthy male subjects (26 ± 2.3 years) were recruited for the study. All subjects reported to exercise on a regular basis (at least 3 hours per week) and had no lower limb injuries in the preceding 12 months. All subjects gave their written informed consent in accordance to the University of Calgary’s policy on research with human subjects. The study protocol was approved by the Conjoint Health Research Ethics Board at the University of Calgary (REB15-2570).

EMG activity of the vastus lateralis (VL) and vastus medialis (VM) muscles of the right leg was recorded with the new current-based EMG amplifier. The tendons of the two vastii muscles transmit their force to the patella and therefore contribute to the stability of the knee joint. The muscles’ medial and lateral locations create medial lateral forces in the knee joint. Thus, asynchronous activation might cause detrimental shear forces. We selected these two muscles because we think that after an injury or after a fatiguing exercise the body loses the ability to optimally control the muscles in a way that is necessary for a stable and efficient movement. The present study is part of a larger project that aims to understand the effect of altered shear forces in the knee joint that may be caused by a lack of synchronization of the motor units. Therefore, our team performed several studies that contribute to better understand the interplay between these two vastii muscles [11, 23, 24, 29].

The muscle fibers of the vastii muscles display a penniform structure. Therefore, it is difficult to 1) align bipolar electrodes with the direction of the muscle fibers on the skin above this muscle which would be necessary to obtain an optimal measure of coherence [22], and 2) measure monopolar potentials because interspersed potentials cause excessive noise. Because the question of knee stability is highly relevant to living a healthy life, we invested a lot of effort in improving the recording system to the point where we could obtain reliable data that allow us to monitor the effects of the neuromuscular control with enough sensitivity to detect differences that we can interpret.

The location for the Ag-AgCl sEMG electrodes (Norotrode dual electrodes, Myotronics-Noromed Inc., USA) was determined by palpation of the muscles during a leg extension movement. Skin preparation included shaving of the area of interest, light skin abrasion with an abrasive medical tape, and final cleaning with 70% isopropyl alcohol, as recommended by the SENIAM [28]. Before the experiment, subjects were asked to perform several contractions of the vastii muscles to visually ensure that a high quality EMG signal was recorded. Appropriate gain to ensure an output of around ±1 V was set, and EMG recorded for each subject at 2.4 kHz with a 14-bit DAQ card (Biovision, Wehrheim, Germany).

**Fig 2:**
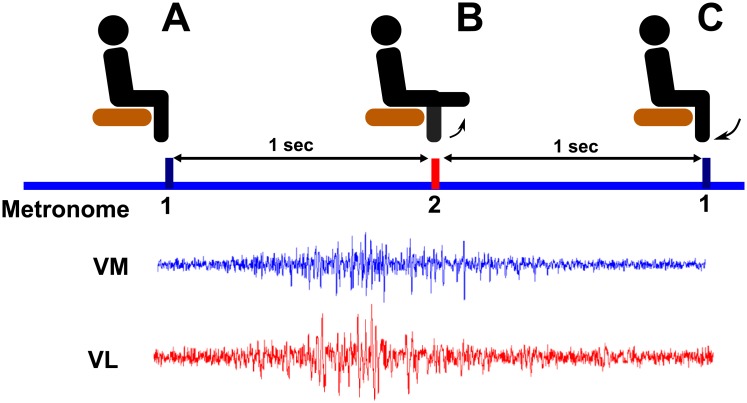
Illustration of the experimental protocol; The blue line marks the metronome cues. (A) cue 1, starting position, 90° knee angle. (B) cue 2, leg extension, 0° knee angle. (C) cue 1, return to starting position, 90° knee angle. The two data traces shown at the bottom are sampled at 2.4 kHz from the VM and VL, respectively.

Subjects attended one experimental session. During the session, the subjects sat on a table that allowed their legs to hang free. They were instructed to perform leg extensions with their right leg at the pace of a metronome. The metronome was programmed to provide two different audio cues at a rate of 60 bpm; the first audio cue instructed the subject to position their leg at a 90° knee angle (Fig 2A), the second cue instructed subjects to have their leg at a 0° knee angle (Fig 2B). This pace leads to an extension-flexion-extension cycle at a 0.5 Hz rate, with distinct phases for the activation of the muscles (Fig 2, bottom). The session consisted of six trials, three control and three biofeedback in a randomized order, with a duration of three minutes per trial. Both conditions were identical, with the exception that for the latter condition, the subjects were provided visual biofeedback about the intermuscular coherence. The biofeedback consisted of a horizontal line representing the value for their coherence of interest, calculated as described in the results section. For the biofeedback trials, subjects were instructed to raise the line without altering the pace or direction of the leg extension movement. The order of the conditions was randomized to avoid biasing effects due to practice or fatigue. After each trial, the subjects were given a two-minute period to stand or walk as desired to avoid potential numbness in their legs caused by the sitting position.

### Intermuscular coherence

As mentioned before, coherence is a measure of the degree of relationship between two time-varying signals as a function of frequency. It is usually presented as magnitude-squared coherence (MSC), a real-valued function defined in Eq 3. To recall, our objective was to test if the intermuscular coherence can be voluntarily increased though a visual biofeedback system. As described in the experimental protocol (methods section), the time series we are interested in are the EMG of VL and VM. Because EMG activations only occur at certain times during the leg extension/flexion protocol, we needed to isolate the periods of EMG activity (Fig 2, bottom) to calculate the intermuscular coherence. We calculated the MSC as follows. Consider *k* *N*-point EMG recordings of the activations of VL and VM, denoted by *x*_*i*_(*n*) and *y*_*i*_(*n*), respectively with *i* = 1, 2, …, *k* and *n* = 1, 2, …, *N*. Over the *k* activations, the mean MSC *C*_*xy*_(*f*) between the EMG signals is given by:

$$\begin{array}{c} C_{xy}\left(f\right)=\frac{|S_{xy}{\left(f\right)|}^{2}}{S_{xx}\left(f\right)S_{yy}\left(f\right)} \end{array}$$ (3)

$$\begin{array}{c} S_{xx}\left(f\right)=\frac{1}{k}\sum_{i=1}^{i=k}X_{i}\left(f\right)X_{i}^{*}\left(f\right) \end{array}$$ (4)

$$\begin{array}{c} S_{yy}\left(f\right)=\frac{1}{k}\sum_{i=1}^{i=k}Y_{i}\left(f\right)Y_{i}^{*}\left(f\right) \end{array}$$ (5)

$$\begin{array}{c} S_{xy}\left(f\right)=\frac{1}{k}\sum_{i=1}^{i=k}X_{i}\left(f\right)Y_{i}^{*}\left(f\right) \end{array}$$ (6)

Where *S*_*xx*_ and *S*_*yy*_ are the mean auto-correlation spectra of the power spectral densities of the EMG of *k* VL and VM activations, respectively. *S*_*xy*_ is the mean cross-correlation spectrum of the EMG of *k* activations of VL and VM. *X*_*i*_(*f*) and *Y*_*i*_(*f*) are the N-point discrete Fourier transforms of *x*_*i*_(*n*) and *y*_*i*_(*n*), respectively. The asterisk symbol (*) denotes the complex conjugate. The resulting MSC is a frequency dependent signal that varies between 0 (i.e. no correlation) and 1 (i.e. perfect correlation) [19].

### Data processing

Data were processed using Matlab 2016 (Mathworks Inc., Natick, MA); this included noise removal, identification and selection of EMG activations, and coherence calculations. First, each channel of the raw EMG signal was filtered for 60 Hz power line noise using a line-frequency averaging method [25]. This method allows the removal of 60 Hz power line noise without altering the phase or inducing a notch effect into the signal. For the selection of EMG activations only the VL EMG activity was used; the VM activity occurs with a similar timing. The filtered signal was passed through a wavelet filter to determine the peaks in the EMG power (i.e. the time points when the muscle activity was strongest) [30]. The wavelet filter used in this study consisted of 11 non-linear wavelets. The power between the 3^rd^ and 11^th^ wavelet, with center frequencies of 37.7 and 395.4 Hz, respectively, was obtained. The sum of the powers across these frequency bands yields the total power, which represents an envelope of the raw EMG that we believe is important for obtaining activation patterns of the muscle. The local peak of the envelope was selected as the mid-point of the EMG activity windows, a bin of 2048 data points (i.e. 850 ms) was selected as the active EMG signal. We denote these data as a single EMG activation. The size of the bin was chosen as it covered the majority of the EMG activation bursts for one leg movement. Coherence between concurrent activations was calculated between the i^th^ VL and VM activations. Coherence between subsequent activations was calculated as the coherence between the i^th^ VM activation and the (i+1)^th^ VL activation. Lastly, a coherence between randomly selected activations was calculated by randomizing the order of the activations in VL.

The statistical significance threshold for intermuscular coherence was calculated as 1 − (1 − *α*)^1(*L*−1)^ [19], where: *α* is the desired confidence level (i.e. 95%), and L is the number of disjoint sections averaged for the coherence calculation (i.e. 90 activations per trial). This threshold was used to identify a frequency range of interest, where the intermuscular coherence showed significant values. A parameter, coherence of interest, was defined as the area under the *C*_*xy*_(*f*) curve within the frequencies of interest, normalized by the area under the perfect coherence (i.e. *C*_*xy*_(*f*) = 1), between the same frequency limits. This coherence of interest was used as the indicator for the visual biofeedback, and displayed to the subject during biofeedback trials. For the real-time display, a sliding window of 10 muscle activations (i.e. *k* = 10) was used to calculate the coherence of interest. Ten activations were chosen as there is a trade-off between the refresh rate of the biofeedback and the coherence estimate. The former needs as few activations as possible, while the latter converges with the number of sequences averaged for the coherence calculation. The number of activations was chosen by a process detailed in [31]. That study looked at the cumulative coherence of the vastii muscles during a cycling protocol. Considering 20 activations at a time reduced the change in cumulative coherence to 10%, that is the coherence changed by less than 10% when 21 or more activations were considered rather than 20. For the present study, we decided to set the threshold to 5%, and assumed considering 10 contractions at a time would suffice. A post-hoc analysis of the cumulative coherence of the control trials of all subjects was performed to test our assumption. To test the hypothesis that the coherence of the vastii muscles can be increased by voluntary commands with a visual biofeedback, all the activations within a trial (i.e. *k* = 90) were used to calculate the coherence of interest.

For each activation, the EMG power of both muscles, as well as the active time (defined as the time that the EMG amplitude was above 30% of the maximum value) were calculated. Additionally, the time between the peaks of EMG activation was calculated.

Statistical analysis was performed with IBM SPSS statistics 24 (SPSS Inc., Chicago, USA). For both biofeedback and control conditions, the mean and standard error of the mean (SEM) of the EMG power, active time, time between EMG activations, and average coherence of interest for the concurrent, subsequent, and randomized activations was computed for each subject. Each variable was evaluated regarding normality with a Shapiro-Wilk test. If the variable was considered to have a normal distribution with the Shapiro-Wilk test, a paired sample t-test or two-sample t-test was performed accordingly. If the variable did not have a normal distribution, a Wilcoxon signed-rank or Wilcoxon rank sum test was done.

## Results

### Hardware characterization

**Fig 3:**
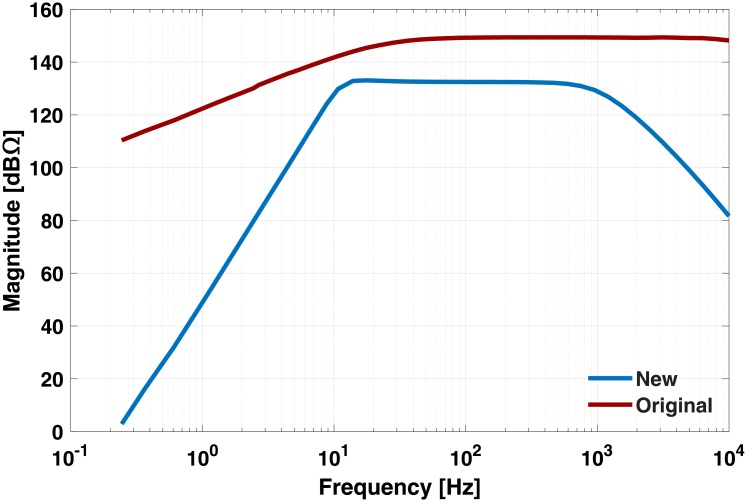
Measured frequency response of the new and original amplifier.

The frequency response measurements show that the new amplifier without the isolation module has a flat (±0.5 dB) magnitude response in the 13 to 500 Hz frequency range (Fig 3). For comparison, the transfer function of the original amplifier is also shown. The input referred noise of the amplifiers for the frequencies between 1 Hz and 10 kHz are 10.7 pA_RMS_ and 12.7 pA_RMS_ for the new and original amplifier, respectively.

**Fig 4:**
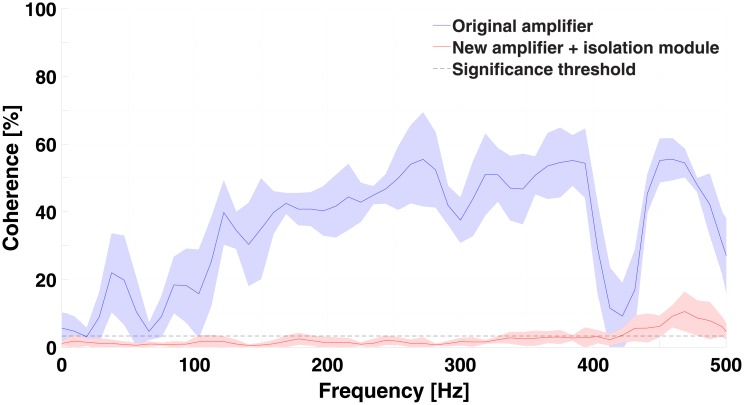
Mean ± SEM of the raw intermuscular coherence of the left and right biceps with left biceps activation only (*N* = 6).

Intermuscular coherence between the activated left, and relaxed right bicep, was calculated using the original amplifier without the isolation module, and the new amplifier with the isolation module are shown (Fig 4). The coherence using the original amplifier exceeds the significance level across all frequencies whereas that using the new amplifier with the isolation module remains below significance.

### Experimental data

**Fig 5:**
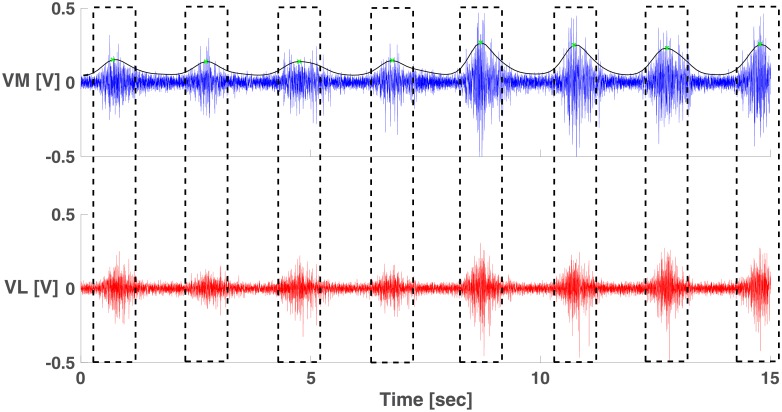
60 Hz filtered VM EMG with wavelet filter envelope (top), and 60 Hz filtered VL EMG (bottom). Traces show EMG data from one subject. Envelope output of the wavelet filter (black trace), midpoints (green dots), and windows (dashed boxes) used for coherence calculation are also shown.

Fig 5 shows the 60 Hz filtered EMG data obtained during a representative trial. Overlaid on top is the envelop output from the wavelet filter and the detected midpoints. The black boxes indicate EMG activations used for calculating the coherence (2048 data points).

**Fig 6:**
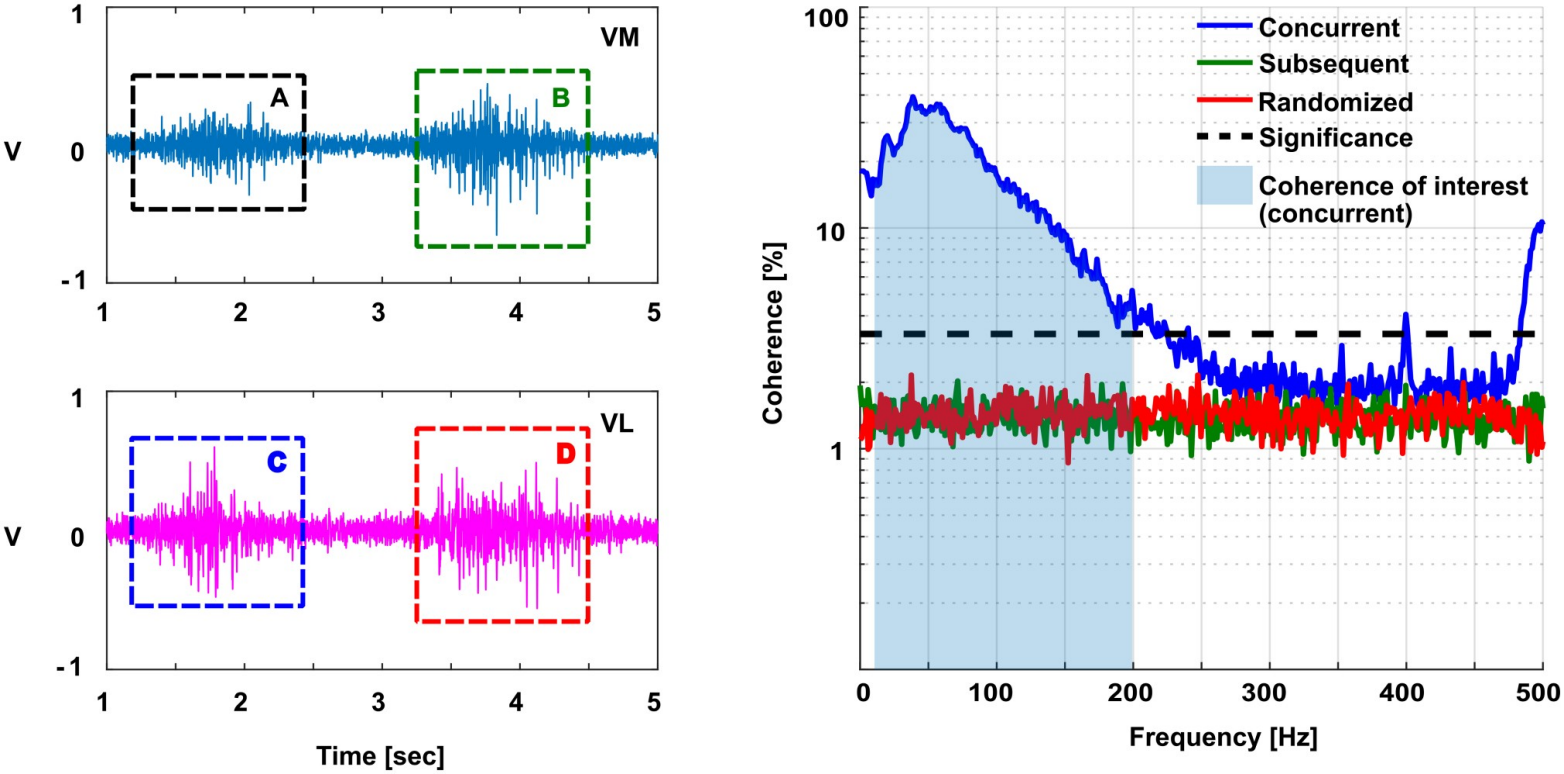
EMG of concurrent activation pairs (A, C and B, D) and EMG of subsequent activations (A, D, and B, C) used to calculate intermuscular coherence (left). Coherence of concurrent (blue), subsequent (green), and randomized (red) activations of one trial (*N* = 90 activations) (right).

Fig 6 shows two consecutive activations of VL and VM during a representative trial; activations A, C and B, D are concurrent; activations A, D and B, C are subsequent. The average MSC calculated from 90 subsequent activations is referred to as the subsequent activations coherence (Fig 6, right). The figure also shows the MSC calculated from 90 concurrent activations. The concurrent activations coherence is higher than the level of significance in the 10 − 200 Hz range; this range was used to calculate the coherence of interest. This is shown as the shaded area normalized by the area of the box representing perfect coherence (i.e. *C*_*xy*_ = 1 from 10 to 200 Hz).

**Fig 7:**
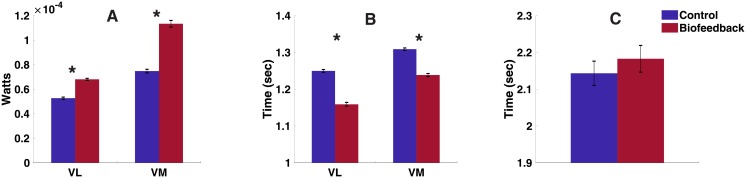
Mean ± SEM of (10 subjects, 6 trials, 90 contractions per trial): (A) EMG power (B) active time, and (C) time between peaks of EMG activation. The asterisk symbol (*) denotes statistical significance (*p* < 0.05).

According to the Shapiro-Wilk test, the EMG power, active time of EMG, and time between contractions were not normally distributed (all, *p* < 0.01). Thus, statistical analysis was carried with a Wilcoxon signed-rank test. Fig 7A shows the mean ± SEM of the EMG power, of VL and VM. This variable showed a significant increase (*p* < 0.001) for both VM and VL in the biofeedback condition. Fig 7B shows the mean ± SEM of the active time of the EMG. The active time showed a significant decrease (*p* < 0.001) for both VM and VL. Lastly, Fig 7C shows the time between EMG activations (mean ± SEM) which was not significantly different between the conditions (*p* = 0.42).

### Voluntary modulation of coherence with biofeedback

**Fig 8:**
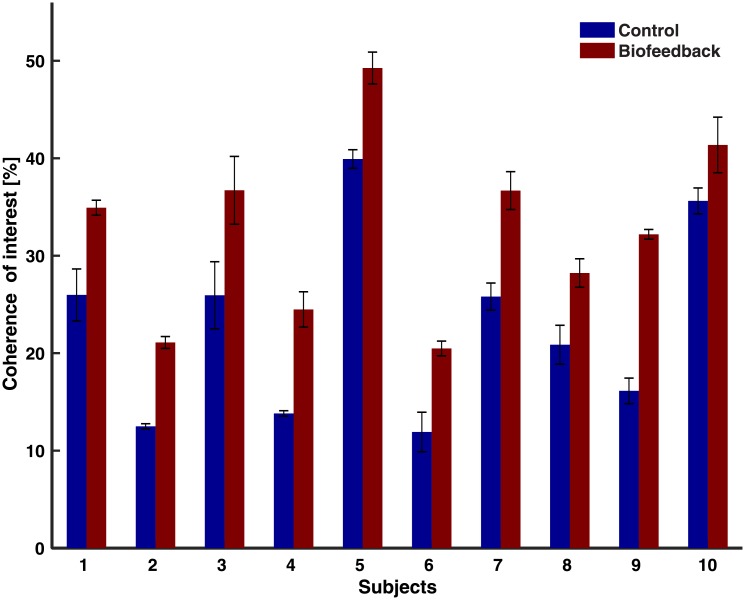
Mean ± SEM (3 trials per condition) coherence of interest.

The cumulative coherence calculation showed that, for the leg extension movement, there is < 1% change in the coherence when using 10 or more activations. The results of the coherence of interest show that all subjects increased their coherence of interest in the biofeedback trials compared to the control (Fig 8). The coherence of interest shows an increase of 25 ± 4.2% (mean ± SEM). We failed to reject the null hypotheses for the Shapiro-Wilk test for normality (*p* = 0.22), thus, the data is assumed to be normally distributed. A paired t-test across conditions shows a significant increase in coherence when comparing the biofeedback to the control condition (*p* < 0.001).

## Discussion

The purpose of this study was to test whether intermuscular coherence of the vastii muscles could be voluntarily increased through visual biofeedback. To achieve such purpose, we developed a visual biofeedback system consisting of an updated version of the current-based EMG amplifier, and developed software for calculating and displaying intermuscular coherence.

### Current-mode EMG acquisition

Our results show that the new amplifier has a flat frequency response in the 13 − 500 Hz region (Fig 3); the difference in the magnitude of the frequency responses is due to the larger TIA gain resistor of the original (2.2 MΩ) compared to the new amplifier (500 kΩ). The original amplifier did not have a low-pass filter; thus, high frequency noise can corrupt the signal of interest. The original amplifier did not have a high-pass filter, thus it needed capacitive coupling with EMG electrodes to reduce low frequency noise. This approach is dependent on skin preparation and the subject, and could be insufficient for a variety of experimental scenarios [24]. The new amplifiers’ high-pass filter is less susceptible to low frequency noise caused by motion artifacts or drift of the signal because of the electrode-skin interface. Regardless of these technical improvements, both amplifiers have a monopolar configuration, thus, external noise in the region of interest (e.g. line frequency noise at 60 Hz) is amplified and corrupts the EMG. Therefore, this configuration still requires digital filters to eliminate any common-mode noise. Future work should focus on developing a system that is more resistant to this noise without losing the current-based methodology. A good approach for this could be a differential current amplifier that benefits from the common mode rejection of an IA.

The results for the isolation module test show that the isolation module can isolate signals from different muscles during simultaneous recordings (Fig 4). The test performed involved the left and right biceps brachii muscles. We used these muscles because it is easy to contract only one biceps muscle at a time. We expected non-significant coherence between independent biceps activations. If the coherence increases over the significance threshold during independent activations, we could interpret this as the presence of cross-talk between the signals. Cross-talk would not allow us to perform any of the visual biofeedback experiments. This cross-talk is thought to be caused by a difference in the impedance of the muscle and ground electrodes and not the muscle in question [24]. Based on this, we believe that our results will remain similar if other muscles were chosen. Using the isolation module, the intermuscular coherence decreased to non-significant values. This can be interpreted as the signals not being related to one another. In the other case, the original amplifier without the isolation module clearly shows high intermuscular coherence, due to cross-talk. This is in accordance with previous experiments with the current-based amplifier [24]. Future work should consider other solutions to simplify the setup while maintaining the isolation. Digitization of the signal within the EMG amplifier module itself should be implemented to improve upon the design presented here. This digitization method has been used in other EMG devices with satisfactory results and increased resistance to external noise [32].

### Voluntary control of coherence with biofeedback

Experimental data shows that for the biofeedback condition, subjects have significantly higher EMG power and shorter activation times (Fig 7A and 7B, respectively). Despite this difference in performance, the movement remained the same (i.e. the time between contractions remained the same across conditions) (Fig 7C). The subsequent intermuscular coherence is similar to the randomized intermuscular coherence across all frequencies, however, the concurrent intermuscular coherence has significantly higher values between the frequencies of interest (i.e. 10—200 Hz) (Fig 6B). This suggests that the concurrent EMG activations share a higher common input than the subsequent or randomized ones.

Additionally, there is a significant increase in the concurrent intermuscular coherence for the biofeedback condition. One could argue that the increase in intermuscular coherence could be because more MUs are being activated, based on the increase in power. However, if the increase in concurrent intermuscular coherence was solely due to the random activation of more MUs’ (which likely happened due to the increase seen in EMG power, Fig 7A), the intermuscular coherence for the subsequent activations would have also shown an increase, which was not the case (Fig 6). Thus, it seems that for the biofeedback condition, the muscles have an increased common input. This could be explained by the clustering effect shown in recent work that suggests that with a shorter activation period during a dynamic contraction, MUs activate in clusters that share a common input [13]. Our experiments show that, to increase the intermuscular coherence during the biofeedback condition, subjects performed a shorter contraction with higher EMG activity. Thus, by constraining the time during which the muscle is active, MUs are required to cluster their activations increasing the intermuscular coherence.

Previous research speculated that intermuscular coherence is controlled by the central nervous system, and it can be modulated by changing the common input of the motor units of the individual muscles [33]. Our results support this speculation. With the appropriate feedback, one is able to change the common input of the motor units [23]. The implications of voluntarily increasing intermuscular coherence have not yet been tested. One study has looked at the difference in intermuscular coherence of the hand muscles between skilled music players and weightlifters [34]. It showed that weightlifters seem to have a higher intermuscular coherence than skilled music players. This could be because musicians require a higher degree of independence in the motor control of their finger whereas weight lifters usually require a higher force generation, which can be obtained by coordinating and synchronizing the muscles. Thus, synchronization of motor units of different muscles seems to be a basic feature of motor control to generate force, especially during a dynamic motor task [21, 23, 24]. We speculate that the biofeedback system presented here could be applied for rehabilitation purposes, where, after a surgery or traumatic event, the body is not able to control the muscles in a coherent manner anymore. One example of this is the rupture of the anterior cruciate ligament (ACL) in the knee; we speculate that the lack of optimal synchronization could result in shear forces and compensations that have negative effects on the cartilage of the knee joint. In fact, it has been shown that such medial lateral shear forces could contribute in addition to the larger co-contraction to the deterioration of the joint after an ACL reconstruction injury, which over time, leads to osteoarthritis [35].

The experiment presented here focused on the intermuscular coherence between the vastii muscles. Studies have looked at the intermuscular coherence of other muscle groups, for example, hand muscles [34], and gastrocnemius [22]. However, the experiment presented here is, to the best of our knowledge, the first attempt to use the intermuscular coherence as a biofeedback signal. It would be of great interest to further research if the results presented here can be replicated in other muscle groups and movements. We speculate that with an appropriate training paradigm, most skeletal muscle groups that are voluntarily activated could be trained to increase their coherence.

Since our experiment is the first experiment of its kind to look at whether one can voluntarily modulate coherence, we decided to use a wide frequency band rather than multiple narrow bands. However, based on [16], we believe that a future experiment could be designed to investigate voluntary intermuscular coherence changes in multiple narrow frequency bands. One could speculate that narrow bands, for instance 10 to 30 Hz, 30 to 50 Hz (Piper frequency) 50 to 100 Hz (majority of muscle fibers) and one around 175 Hz (fast conducting muscle fibers). There is a hint towards these bands in Fig 6, however not sufficiently clear to point to these bands in this manuscript. We resolved them more distinctively in another study [11].

To date, central fatigue of muscle activity refers predominantly to the inability to keep up with exercise performance, and cannot be explained by peripheral factors that affect muscle function [24]. An alternative sign of fatigue could be a decline in the ability of the central nervous system to further support optimal coherent activation of the vastii muscles. As mentioned before, preliminary results in our laboratory have shown that intermuscular coherence decreases to the progression of muscle fatigue during squatting movements [24]. As fatigue increases, intermuscular coherence tends to decrease. It has recently been shown that during cycling, intermuscular coherence increases with power output [36]. Alterations in the neuromuscular strategy (i.e. muscle activation pattern and power contribution of individual muscles) were evident as the power output increased [36]. However, the relation between intermuscular coherence and fatigue during cycling or other sports activities has not been tested. If decreasing intermuscular coherence is a causative factor of fatigue, we believe our biofeedback system could be used to train subjects to increase endurance during fatiguing exercises. This possibility warrants studies that may show a significant impact in sports activities where endurance plays a key role in the performance of athletes. Additionally, our experiment tested only the instantaneous effects in the intermuscular coherence caused by the visual biofeedback. Further research should be done to investigate any long-term effects (e.g. neuroplasticity) of such methodology. Neuroplasticity effects are an important factor of long-term rehabilitation, for example after a stroke [37]. If the methodology induces long-lasting recovery of the neurological control of muscles, it could be used for rehabilitation purposes.

The main limitation of our experimental protocol was the limited measurement of the movement. The subjects were asked to perform the same movement based on the metronome sound. Although the movement was visually inspected to remain consistent, the instantaneous velocity or extension/flexion angles of the leg movement were not measured. Our results could have benefited from such measurements to determine the movement strategies that the subjects used to increase the intermuscular coherence. However, neither the subjects nor the researchers were aware of any obvious change of the movement; this is also supported by the time between contractions remaining the same across conditions (Fig 7C). Additionally, a window of 10 muscle activations was chosen to calculate the coherence of interest during the biofeedback experiment. As mentioned before, there is a trade-off between the refresh rate of the biofeedback signal and the coherence estimate. We arbitrarily chose a 5% threshold for the change in cumulative coherence, and used 10 activations to achieve the threshold. While this was not tested during the study, post-hoc analysis showed considering 10 activations reduced the change in cumulative coherence to < 1%. Further study is required to inform the change threshold, and the number of activations needed to achieve it, that would optimize the trade-off between coherence estimate and biofeedback refresh rate. This can make the biofeedback process much more intuitive for the user, and thus, yield better results.

## Conclusions

We presented a biofeedback system that uses intermuscular coherence of the vastii muscles as the main feature. To optimally monitor such change in coherence, a multichannel current-based EMG system with an isolation module was developed. A software to monitor the instantaneous intermuscular coherence was developed; the coherence was shown as a visual biofeedback to the subjects. The results showed that the 10 recruited subjects could voluntarily increase their level of coherence with the use of the visual biofeedback. To the best of our knowledge, this is the first study to indicate that one can voluntarily increase intermuscular coherence, and thus synchronization of the vastii muscles. We are confident that biofeedback systems based on intermuscular coherence can have applications in rehabilitation and training.

## Supporting information

S1 File Frequency response, cross-talk coherence, time-domain analysis, biofeedback coherence. (XLSX)
